# On the Tacit Aspects of Science Pedagogy in Higher Education

**DOI:** 10.3389/fpsyg.2017.00656

**Published:** 2017-05-03

**Authors:** Ramakrishnan Sitaraman

**Affiliations:** Department of Biotechnology, TERI UniversityNew Delhi, India

**Keywords:** implicit knowledge, apprenticeship, history of ideas, non-formal learning, history of science, affective learning, higher education, higher-order thinking skills

## Abstract

In this article, we examine the concept of tacit knowledge and its implications for science education. We suggest that the history of scientific ideas and the personal nature of learning imply that higher education in scientific fields, wherein the generation of new knowledge, insights and understanding is paramount, would greatly benefit by acknowledging the irreducible role of the non-formal and the incidental in scientific innovation and advances.

What a misfortune it is that we should thus be compelled to let our boys’ schooling interfere with their education! – *Post-prandial Philosophy* by Grant Allen (1894) .

## Introduction

The idea of ‘tacit knowledge’ has its origins in the writings of Michael Polanyi, elaborated in detail in his book *The tacit dimension* (Polanyi, 1967). In his book, he suggested that the successful performance of tasks requiring multiple skills, specifically scientific inquiry, requires not only a thorough knowledge of the procedures and techniques involved in a purely objective sense, but also a personal component of insight, experience and even creativity that cannot be not easily explicated by the practitioner himself. This is summarized in his maxim “We can know more than we can tell.” The presentation of this issue by Polanyi endows the process of scientific innovation with an aura of both mystery and uncertainty. Polanyi’s idea of ‘tacit’ knowledge took root in his philosophical opposition to the Soviet ideological position enunciated by Nikolai Bukharin that scientific inquiry would automatically proceed in accordance with the exigencies of the 5-year plans formulated by the socialist state (Polanyi, 1967). As Schmidt (2012) points out perceptively, Polanyi’s ideas were formulated with intention of insulating ‘pure’ science from notions of state control. This concern was expressed by positing a component of skilled performance that could not, by definition, be codified in any objectively meaningful way. By endowing science and scientific advancement, especially in ‘pure science,’ with an irreducibly personal and vital component, Polanyi seems to indicate the eventual inability of state-mandated regimens to encourage, and by implication completely account for, scientific innovations. In pedagogy too, a personal and vital component is present in the acts of both teaching and learning that contributes an intangible, but nevertheless valuable aspect to the lived experience of both the teacher and the taught.

## The Reinterpretation of the ‘Tacit’ By Nonaka and Takeuchi (1995)

In industrial settings, the tacit dimension was reinterpreted with specific reference to ‘skilled performance’ and efforts were made through close observation of the master-craftsman-apprentice relationship to explicate what had remained implicit. Analogously, in both science and the pedagogy of science, the transfer of skills from teacher to student is also of undeniable importance. The very notion of specifying ‘learning outcomes’ implicitly indicates that a transfer of codifiable skills and knowledge is what is normally expected in the pedagogical context. To summarize, there are two aspects to the pedagogy of science: the effective transfer of knowledge and skills from teacher to taught, and a more intangible aspect of innovation that comes only by an uncertain combination of deep knowledge, personal involvement, intuition and creativity. It is to these issues that we now turn.

When the literature on tacit knowledge is searched for studies about the tacit components of knowledge in attaining preferred outcomes, a clear dichotomy is observed between the ‘pure’ and the ‘applied’ sciences, mirroring Polanyi’s original concern about safeguarding pure sciences from state control. The representation of studies is greatly skewed toward engineering and medicine, i.e., applied and professionalized fields of scientific endeavor. Thus, pure science practitioners have, by and large, not investigated the role and impact of tacit knowledge in their field to the same extent that their colleagues in the applied sciences have. This is understandable because the applied sciences have to grapple with the issue of effective skill transfer and therefore, productive apprenticeship, practically on a daily basis. Therefore, the fields of engineering and medicine are acutely conscious of the need to codify (and thereby preserve by depersonalizing) useful and actionable knowledge, and the repeated requirement for heuristics in the sense of ‘rules of thumb’ to enable reliable judgments during problem-solving. We suggest that the vocational narrative of ‘pure’ science supports self-motivated and disinterested inquiry into phenomena that de-emphasizes the codification of ‘best practices’ for innovation. The idea of serendipity, of “chance favoring the prepared mind’ (*vide* Louis Pasteur) is deeply embedded and highly appreciated in this ideational framework, which is not conducive to the kind of efforts that Nonaka and Takeuchi make in order to render explicit formerly tacit knowledge. Thus, the unstated ‘tacit’ that requires ‘chance’ to meet the ‘prepared mind’ permits the pure sciences to ‘tacitly’ dispense with the need for codification of their ‘best practices’ simply because they don’t exist in an objective sense.

As in the case of Polanyi responding to a contention by Bukharin who was grounded in Soviet ideology, the writings of Nonaka and Takeuchi also had a reactive context (Nonaka and Takeuchi, 1995). As their book is summarized by the publisher Oxford University Press:

“How has Japan become a major economic power, a world leader in the automotive and electronics industries? What is the secret of their success? The consensus has been that, though the Japanese are not particularly innovative, they are exceptionally skilful at imitation, at improving products that already exist. But now two leading Japanese business experts, Ikujiro Nonaka and Hiro Takeuchi, turn this conventional wisdom on its head: Japanese firms are successful, they contend, precisely because they are innovative, because they create new knowledge and use it to produce successful products and technologies. Examining case studies drawn from such firms as Honda, Canon, Matsushita, NEC, 3M, GE, and the U.S. Marines, this book reveals how Japanese companies translate tacit to explicit knowledge and use it to produce new processes, products, and services” (see OUP website^1^).

The last statement is especially important, that Japanese companies owe their effectiveness and ascendancy to their ability to ‘translate tacit to explicit knowledge.’ Incidentally, it seems to indicate that other organizations could achieve similar excellence by following suit. But note that the case studies are mostly concerned with industries, and definitely not with educational institutions. Indeed, the element of depersonalization consequent on making tacit knowledge explicit arises precisely because industry needs to have well-defined and reproducible means and methods of production, and can ill-afford to allow useful knowledge to remain the preserve of a few ‘gifted’ employees in the form of tacit knowledge. Uncodified and non-verbal knowledge is therefore a genuine problem for manufacturing processes. This is because the absence of such knowledge is never observable *a priori* but can at best be inferred only *a posteriori*, after the manifestation of anomalous outcomes that, in extreme, may even be physically dangerous, e.g., an incorrectly executed step or omitted precaution in the usage of heavy machinery.

This analysis of the relative importance of codifying tacit knowledge in the ‘pure’ and ‘applied’ sciences is itself founded on an assumption that we now make explicit: That there is an objective and therefore observable, dichotomy between the ‘pure’ and the ‘applied.’ The prevailing narrative assumes that the ‘pure’ is worth pursuing not only for its own sake, but because it may eventually lead to ‘applications’ in the real world. Thus, there is an element of utilitarianism in the funding of ‘pure’ science that is intermittently highlighted, especially in situations where funding limitations become an overriding concern, e.g., the Large Hadron Collider (Llewellyn Smith, 2010). Likewise, biologists have used the phrase ‘bench to bedside’ to indicate the ultimate utility of their investigations that are manifestly undertaken initially out of innate curiosity about the nature of living organisms. However, the actual history of science is replete with instances of cross-fertilization between the administratively convenient compartments titled ‘pure’ and ‘applied’ (Sitaraman, 2012). Given the foregoing discussion, educators in the scientific field would be well-served by investigating the actual nature of scientific innovation.

## The Narrative Fallacy^2^ and the Educator

Institutions of higher education, by which we mean those that emphasize research and innovation within the student body under the guidance of faculty members who are trained practitioners, are expected to not only impart skills that enable students to effectively undertake technical procedures relevant to their field of study. Supported as they are by infusions of a combination of public or private funds, they are also expected to continuously contribute to the creation of new knowledge, novel interpretations of existing knowledge, and synthesis of multiple, often disparate, strands of information leading to a greater understanding and application of the topic at hand. Thus, the emphasis in higher educational institutions shifts decisively toward higher order thinking skills as stated in Bloom’s taxonomy (Bloom, 1956), with the seldom-stated, but nevertheless real hope that the ‘next great invention or discovery’ would emanate from the ranks of their faculty and students. For example, the British physicist Sir Peter Higgs, Nobel Laureate in Physics (2013) stated in an interview to *The Guardian* that the authorities of Edinburgh University retained him on the rolls in spite of the paucity of ‘regular’ research publication in the hope that he might win a Nobel prize based on his 1964 work on the eponymous Higgs boson (Aitkenhead, 2013).

This institutional drive to attain and maintain high rankings in the highly competitive area of university education creates a certain tension between researcher and institution based on what may be deemed ‘important’ or ‘cutting-edge’ in science at a given time, but does not explicitly acknowledge the process by which science has historically evolved. Thomas Kuhn in his 1962 study of the sociology of science divided the scientific enterprise not into the familiar categories of ‘pure’ and ‘applied’ but into ‘normal’ and ‘revolutionary’ (Kuhn, 2012). We suggest that normal and revolutionary are not only qualitatively distinct categories in terms of the objective impact of the scientific advance in question. Rather, these objective attributes are additionally indicative of underlying cognitively distinct categories as well. The reason for such an inference is based on the nature of activities involved in each domain.

In Kuhn’s framework, the domain of normal science is the incremental addition of detailed knowledge and insights based on the prevailing consensus by a process of ‘puzzle-solving.’ Any hypotheses that are framed are rooted in accepted general principles and models. Notably, this type of scientific endeavor is clearly amenable to both institutionalization and professionalization. Almost by definition, this does not lead to the kind of ‘great’ advances and inventions that are highlighted in a narrative history of science because, while the incremental accretion of knowledge in a field may be of immense interest to insiders, it does not necessarily arrest the attention of either the student or the public at large. According to Kuhn, major advances and innovations occur almost organically when the accumulation of non-conforming information crosses a critical threshold that he termed as a ‘crisis’ in the discipline. The idea of a disciplinary crisis is certainly an apt metaphor in some historical instances. For example, it accurately reflects the kind of experimental problems that laid the foundations for the historical development of Einstein’s special theory of relativity and quantum mechanics. However, it is not readily applicable in other cases, for example, Gregor Mendel’s work (1866) on inheritance in pea plants and his theory of ‘genes,’ that were not precipitated by any recognition of a pre-existing ‘crisis’ in the field of biology. Instead, the theory of dominant and recessive genes was proposed as a model to account for the relative proportions of offspring displaying distinct external features or phenotypes when pure-breeding parents of different phenotypes were crossed. Therefore, textbooks repeatedly emphasize that Mendel was ‘ahead of his times,’ which statement implies that he was not responding to any disciplinary crisis. Mendel published his work 7 years after Charles Darwin published his celebrated *Origin of Species* in 1859. Very importantly, though the *Origin* went through five more editions that involved significant revisions under Darwin’s supervision, the last being published in 1872, Darwin labored unaware that Mendel’s postulate of ‘genes’ could provide a crucial concept with explanatory power for the actual mechanism of both heredity and evolution. It was only with the “modern synthesis” initiated at the beginning of the twentieth century by Hugo de Vries that the relationship between genes and organismal evolution was perceived.

The history of science therefore contains not only celebrated accounts of serendipity, improbable coincidences and idiosyncratic insights, but also less frequently mentioned instances of missed chances and lost time. We therefore suggest that the actual history of science is less amenable to ‘narrative-building’ than we would like. This, in turn, implies a certain loss of control of processes and events, which is worrying in the professional realm wherein employees and organizations alike are expected to direct efforts toward repeatedly and reproducibly achieving (often monetarily) tangible outcomes. Institutional mechanisms are sought to be formulated to encourage scientific advances that have historically defied the reduction to a ‘standard narrative’ or, in corporate parlance, a ‘standard operating procedure’ (SOP). For example, Nonaka and Takeuchi develop the idea of a ‘hypertext’ organizational structure that would be conducive to the creation and operationalization of formerly tacit knowledge, by being able to flexibly alternate between predictable and efficient bureaucratic processes and a problem-based task force approach (Nonaka and Takeuchi, 1997). However, the present work is more focused on the individual educator, and we suggest that this undoubtedly crucial issue of organizational facilitation for the exercise of individual abilities deserves a discussion on its own.

The interaction of a scientist with the environment and his/her simultaneous usage of prior experience and cognitive ability is of paramount importance in making those major advances that are eventually incorporated into ‘narratives’ of the history of science. The requirement for such a narrative is an expression of the deep-seated human need for order, coherence and certainty in a world of randomness and uncertainty, leading to a cognitive (but unconscious) highlighting of seeming certitude and retrospective predictability. A narrative is definitely valuable, as it may enable us to deduce general principles from seemingly unconnected pieces of information, but it overstays its welcome when it becomes an end in itself. As educators, we also unconsciously subscribe, *mutatis mutandis*, to some version of this narrative fallacy when undertake comparisons of pre- and post-test scores after devising some creative educational intervention. We would not analyze these results if we did not really believe that it would eventually lead to something beyond the attainment of the immediate learning outcome of interest. Specifically, we hope that we are thereby enabling not only the effective transfer, retention and application of new concepts and information, but also empower our students to “learn how to learn” the selfsame concepts, procedures and ideas. While this student-centered view is unexceptionable, it also begs the question of the emphasis on objectively measurable outcomes in our work of teaching and mentoring. One suspects that, once an objective measure is emphasized at an institutional level, teachers (like all other professionals conscious of their careers) react in accordance with Goodhart’s ‘law’ that suggests that a metric ceases to be useful once it is adopted as a target, because people begin to try and manipulate it to their advantage. The San Francisco Declaration on Research Assessment, 2012 suggesting that journal metrics and other measures of ‘prestige’ not be used as *de facto* markers of research excellence is an indication of how objective and quantitative metrics, while convenient and efficient from an administrative perspective, may not truly serve the cause of scientific advancement.

## The Uncertainty of the Tacit Dimension

From the history of science and the widespread professionalization of scientific research, we may well wonder if the entire issue of communicating tacit knowledge, thereby making it manifest and codifiable is in fact an admission of discomfort with uncertainty that humans generally have. After all, insurance policies address themselves to the one uncertain certainty we all agree on: Death is inevitable, yet its precise timing is unpredictable. As Michael Eraut (Eraut, 2000) points out perceptively, professional settings necessarily prompt descriptions that emphasize predictability and control in order to inspire confidence in the listener. Analogously, in the realm of science pedagogy too, there is a clear and ongoing attempt to improve teaching, and thereby learning outcomes, preferably in terms of reproducible and quantifiable metrics. Even subjective issues like interest and presentation style are often sought to be quantified by asking respondents to estimate scores on a numerical scale, which can be treated as a conventionally quantitative measure. If the goal of higher education is to promote research and innovation, it will need to employ approaches that explicitly recognize the uncertainties and risks associated with the successful production and valorization of new knowledge. Indeed, funding agencies often refer to their range of sponsored activities as comprising their ‘research portfolio’ consisting of research programs and individual projects with varying investment, risk and reward profiles. The Science and Engineering Research Board (SERB) of the Government of India has introduced a separate category of extramural grants that are termed ‘high risk high reward’ that is an explicit acknowledgment of this uncertainty (see SERB website^3^).

The tacit is therefore characterized by both uncertainty and a lack of formalism. The dilemma before the teacher is to ensure the adequate availability of opportunities for non-formal learning, but without materially compromising the overall didactic goals and rigor of a given program of study. Given the inevitably personal nature of tacit knowledge, it would help to consciously recognize that learning outcomes are by no means guaranteed, even if they arise in the context of well-controlled and previously tested interventions with tangible results. This is because a change in audience inevitably occurs with successive groups of students having varied abilities and preferences. Rather, the aim of teaching should include not only the attainment of learning outcomes, but the encouragement of deep and introspective engagement with the study material. Thus, the inevitable evaluation strategy may be itself evaluated not only by the improvement of test scores in the conventional sense, but also the degree of introspection and deep learning it fosters. Contrary to positivist notions of improving learning outcomes, such an opportunity would actually result in students realizing that their interests and abilities (as well as the lack thereof) preclude further deep engagement with the subject at hand. It is therefore germane for teachers to accept the personal and subjective nature of the cognitive process that underlies the successful transfer and acquisition of knowledge and skills. The personal value for a student to conclusively recognize that his/her interests and skills lie elsewhere than the subject they have enrolled themselves in is also a valuable learning outcome that materially affects career choices. Thus, teachers and educational institutions would gain much by explicitly recognizing the role of chance and the essential non-codifiability of ‘best practices.’

Another key issue when it comes to application is the actual amount of time a given course or program of study has to devote to develop such valued tacit capacities. It is one thing to accept the principle that non-conventional opportunities for learning have to be accommodated, but quite another to determine what specific proportion of a given course should be devoted to such activities. To this, we propose that the cause is better served by awareness-raising, rather than by codifying a SOP to improve tacit skills. From the viewpoint of learning outcomes, the time investment turns out to be a ‘high-risk investment,’ but one with potentially major payoffs or losses. This has to be balanced with low-risk investments of the conventional kind, such as creatively designed didactic modules, paper presentation and writing, evaluation schemes designed to test for specific learning outcomes (insofar as they can be specified), and so on. Only by having a wide range of approaches can we hope to facilitate, however, imperfectly, the process by which advances in knowledge and innovations in practice occur in the real world.

The foregoing discussion may prompt the accusation that the reduction of humans to black boxes is implicit in the idea of improvements in pedagogy for groups. As educators know from experience, every batch of contemporary university students is a group of very different individuals who have come together by accident as a result of an (hopefully) impartial admission process. Therefore, beyond the fulfillment of the stated ‘minimum eligibility requirements’ for the given course, there is very little such individuals have in common. When careful attention is paid to both the abilities and deficiencies of the individuals who comprise the student body, we find not only those who are genuinely interested or invested in the program, but also those who are not really interested in the subject, but lack an outlet or opportunity to express their other talents and abilities. In higher education it is therefore critical that, in addition to improvement, we also offer opportunities for introspection to our students. Specifically, the didactic and interrogative components of the course should enable them to discover *for themselves* whether they have indeed made the correct choice of a course of study, and whether there is sufficient alignment with their personal career objectives and priorities. This, of course, is impossible unless students also have the chance discover during the process of introspection whether or not their abilities and interests are aligned with the demands of the course at hand. Importantly, while we can learn from failure, we instinctively shrink from the suggestion of failure. This process of learning from one’s mistakes is essential to one’s growth as an individual who seeks new external information and understanding, as well as a critical appreciation of one’s own strengths and weaknesses within the context of personal aspirations. Thus evaluation modules designed by the teacher have, or may acquire, functions in addition to the conventional one of determining student proficiency in a particular subject. It is now to this deeply personal aspect of self-discovery by students that we now turn.

## Effective Pedagogy is Deeply Affective

A very important aspect of pedagogy that is often missed in studies of curriculum improvement is the idea of ‘pleasure in pedagogy.’ In their book *The Slow Professor*, authors Maggie Berg and Barbara Seeber devote an entire chapter to this issue, in which they draw our attention to the issue of affect, when deep emotional involvement with the subject at hand creates a form of pleasure that transcends even the objective fact of grappling with a difficult problem and solving it (Berg and Seeber, 2016). Sir Peter Higgs (quoted earlier) also remarked that he wouldn’t have had the kind of ‘peace and quiet’ that he enjoyed in 1964 in the academic atmosphere of today (Aitkenhead, 2013), indicating that there are other issues at work in producing major advances in scientific knowledge.

In fact, we suggest that, in addition to the satisfaction of expending honest effort at explaining and understanding, there is an additional element of the esthetic that informs such pleasure. Brooks (2009) states in the context of medical education that “True expertise is transmitted not by lectures or textbooks, but by guided practice.” Even the intellectually and physically taxing work of scientific research, with it methodological norms (and strictures) may be valuable to the dedicated researcher in ways that cannot be expressed except perhaps in terms of an esthetic ideal. The objective non-practitioner may, with some justification, feel that “there are better things to do.” The important fact is that a sense of wonder, discovery and esthetic pleasure is what ultimately not only enables, but more importantly, sustains a deep interest in any subject, and amounts to a truly lasting ‘learning outcome’ at the individual level. As we readily confess, it is this attribute that reminds us of the ‘great teachers’ that we have encountered during the course of our own education, even long after we have lost touch with the subject they taught. Perhaps it is this that remains forever ‘tacit’ and yet, powerfully informs the tasks of both the skilled and the learned. In science education, the stress is understandably on measurable outcomes, not only because these are measurable outcomes, but also because the idea of measurement itself is deeply embedded in scientific culture. However, as educators specifically engaged with higher education, we would do well to recognize the irreducibly personal nature of all knowledge that contributes to a satisfying, even memorable educational experience for the student, regardless of the eventual utility of the subject matter in their subsequent career. Finally, we note that Hafler et al. (2011) perceptively point to the lack of acknowledgment of faculty as learners, something that greatly contributes to the dedication and innovation that faculty members are expected to bring to their task, and enriches their own experience of the didactic process.

An additional fact to be appreciated is that humans have instinctively felt at ease with the determinism that a well-formulated dichotomous framework can bring to our understanding of our world and thereby simplify decision-making. Classic examples are “pairs of opposites” such as living and non-living, plant and animal, night and day, likes and dislikes and so on. However, we would like to emphasize that we do not wish to imply a similar (irreconcilable) dichotomy in the case of tacit and explicit knowledge. Rather, we concur with the idea of a continuum of knowledge enunciated by Nonaka and von Krogh (2009) wherein tacit and explicit knowledge are “mutually complementary,” and not objectively separate and mutually exclusive as may be implied by a dichotomous view. Their view that the ongoing dynamic interaction between the two types of knowledge eventually results in knowledge creation provides a valuable conceptual framework for teachers to retrospectively analyze and prospectively plan their academic activities.

Another point that needs to be noted is that the work of Nonaka and Takeuchi as summarized by Stillwell (2003) indicates that a collectivist organizational framework is an underlying assumption in those studies and theorizations. In such a system, the very first process envisaged is socialization wherein “each person’s tacit knowledge is converted to tacit knowledge now also held by other members in the microcommunity.” This is followed by externalization, combination and eventual internalization by other members of the community. The final objective in this context is that the knowledge and proficiency levels be harmonized to the extent possible across the organization. This can, to a great extent, mirror some successful academic processes, such as building a collaborative research team and also carrying out ‘normal science’ at an individual level. However, we feel it does not adequately represent the disproportionate contributions that specific individuals and their insights in often improbable circumstances have historically brought to the process of knowledge creation and innovation resulting in ‘revolutionary science’ (see “The Narrative Fallacy and the Educator”).

Ours therefore is a case for viewing science education as not only a science, but also an art with potentially as many styles as practitioners. Polanyi’s idea of tacit knowledge is a useful concept that *can* vitally and creatively inform our pedagogical efforts, but internalization and expression are highly dependent on the abilities and interests that faculty members bring to their task beyond meeting the essential objective qualifications for their positions. And, just as in art, while proficiency in a variety of pedagogical techniques is a useful, even necessary requirement, it is by no means a sufficient one.

## Author Contributions

RS conceptualized this work, collected references, analyzed information and wrote the article.

## Conflict of Interest Statement

The author declares that the research was conducted in the absence of any commercial or financial relationships that could be construed as a potential conflict of interest.

## Footnotes

1 The knowledge-creating company: how Japanese companies create the dynamics of innovation (1995). Available at: https://global.oup.com/academic/product/the-knowledge-creating-company-9780195092691?cc=in&lang=en&# (accessed March 24, 2017).

2 See (Taleb, 2010).

3 High Risk High Reward. Available at: http://serbonline.in/SERB/HRR (accessed February 27, 2017).

## References

[B1] Aitkenhead D. (2013). *Peter Higgs: I Wouldn’t be Productive Enough for Today’s Academic System.* Available at: https://www.theguardian.com/science/2013/dec/06/peter-higgs-boson-academic-system [accessed February 21 2017].

[B2] Berg M. Seeber B. K. (2016). *The Slow Professor: Challenging the Culture of Speed in the Academy.* Toronto, ON: University of Toronto Press.

[B3] Bloom B. S. (1956). *Taxonomy of Educational Objectives; The Classification of Educational Goals.* New York: Longmans, Green.

[B4] Brooks M. A. (2009). Medical education and the tyranny of competency. *Perspect. Biol. Med.* 52 90–102. 10.1353/pbm.0.0068 19168947

[B5] Eraut M. (2000). Non-formal learning and tacit knowledge in professional work. *Br. J. Educ. Psychol.* 70 113–136. 10.1348/000709900158001 10765570

[B6] Hafler J. P. Ownby A. R. Thompson B. M. Fasser C. E. Grigsby K. Haidet P. (2011). Decoding the learning environment of medical education: a hidden curriculum perspective for faculty development. *Acad. Med. J. Assoc. Am. Med. Coll.* 86 440–444. 10.1097/ACM.0b013e31820df8e2 21346498

[B7] Kuhn T. S. (2012). *The Structure of Scientific Revolutions; with an Introductory Essay by Ian Hacking* 4th Edn. Chicago, IL: The University of Chicago press.

[B8] Llewellyn Smith C. H. (2010). *What’s the Use of Basic Science?.* Available at: http://wwwold.jinr.ru/section.asp?sd_id===94 [accessed February 27 2017].

[B9] Nonaka I. Takeuchi H. (1995). *The Knowledge-Creating Company: How Japanese Companies Create the Dynamics of Innovation.* New York, NY: Oxford University Press.

[B10] Nonaka I. Takeuchi H. (1997). “A New Organizational Structure,” in *Knowledge in Organisations* ed. Prusak L. (Boston: Butterworth-Heinemann) 99–133.

[B11] Nonaka I. von Krogh G. (2009). Perspective–tacit knowledge and knowledge conversion: controversy and advancement in organizational knowledge creation theory. *Organ. Sci.* 20 635–652. 10.1287/orsc.1080.0412

[B12] Polanyi M. (1967). *The Tacit Dimension.* Garden City, NY: Anchor Books.

[B13] San Francisco Declaration on Research Assessment (2012). Available at: http://www.ascb.org/files/SFDeclarationFINAL.pdf [accessed February 27 2017].

[B14] Schmidt K. (2012). The Trouble with ‘Tacit Knowledge’. *Comput. Support. Coop. Work* 21 163–225. 10.1007/s10606-012-9160-8

[B15] Sitaraman R. (2012). From bedside to blackboard: the benefits of teaching molecular biology within a medical context. *Perspect. Biol. Med.* 55 461–466. 10.1353/pbm.2012.0030 23179036

[B16] Stillwell W. D. (2003). Tacit knowledge and the work of ikujiro nonaka. *Tradit. Discov. Polanyi Soc. Period.* 30 19–22.

[B17] Taleb N. N. (2010). *The Black Swan: The Impact of the Highly Improbable*, 2nd Edn. London: Penguin Books, 62–84.

